# Evensong: how the medical humanities can strengthen a patient-centred approach to both physical and mental health conditions

**DOI:** 10.1192/bjb.2021.3

**Published:** 2022-06

**Authors:** Corinne Rowena Dignan

**Affiliations:** St Cadoc's Hospital, Newport, UK

**Keywords:** Education and training, dementia, comorbidity, organic syndromes, stigma and discrimination

## Abstract

The medical humanities may offer an antidote to the unconscious depersonalisation of patients into clinical variables and diagnoses at the hands of physicians, cultivating a patient-centred and individual approach to the management of both physical and mental health conditions. The emphasis on the person behind the diagnosis helps physicians to remain motivated and compassionate in the face of increasing social and organisational pressures that threaten this human connection. As a doctor and the relative of a patient with dementia, I reflect on the way in which poetry has helped to translate my experience as a relative into improving my own practice as a doctor. This article includes one of the poems I wrote during my grandmother's illness to aid reflection on the patient perspective I gained during her time in hospital, and also a brief commentary exploring the influence this process has had on the interactions I now have with my patients.

We all have our stories. Our triumphs and our failures, the people we love, the landscapes we create and the goals we pursue; all those days, from the greatest moment to the most mundane, come together to form the fabric of a life.

## Presenting complaint

One of the great privileges of a physician is that our patients share their stories with us, and our eyes witness the momentous: the first cry of new life, the last sigh as old age departs it, and countless tears, smiles and occasional bursts of anger that our involvement in these stories inevitably brings. During my time as a doctor, however, I have noticed that by assigning the role of patient to these individuals within our own narrative, we often, however unconsciously, deprive them of their individual story. They become lists of vital signs, blood results and diagnostic difficulties, the faces of medical conditions that leap out of textbooks into the hospital beds in front of us, embodying the frailty and mortality that is truly such a small part of who they really are.

## Past medical history

I first became sensitised to this when my grandmother's memory faded with vascular dementia, which followed the stroke she suffered while I was at medical school. Her story was lost to the doctors caring for her on the ward, and the impression that they formed of the truculent and demented old woman who obstinately refused her medication was infinitely removed from the independent, active and astute lady of just a few weeks earlier, the one with a roguish twinkle in her eye who loved to sing, laughed often and always cheated at playing cards. This was compounded by the resistance that we, as her relatives, experienced when attempting to engage with the doctors in our new role as proponents of her story, finding that our attempts at advocacy were rejected as an irrelevant distraction from her medical care.

The condensation of a rich and complex individual into the black and white of a clinical diagnosis does our patients a disservice, but it is a phenomenon that many doctors report in their professional practice. For my grandmother it contributed to delays in her diagnosis, a lack of appropriate stroke rehabilitation, a void of professional support and an inexorable decline into depression, incontinence and pressure ulcers. The strain of increasing service demands without a correlating increase in resources and support stands as an obvious culprit, but the influence of compassion fatigue and the unconscious desire to protect our own sanity and emotions when treatments fail and operations do not go to plan cannot be ignored.

## Investigations and management

Reflecting as both a physician and a relative, I realised that I too frequently overlooked the person behind the confused and wandering patients on my ward; I was also too ready to make assumptions about the quality of life of octogenarians, and I recognised the previously unacknowledged dread of the demands of relatives during visiting time while my pager chimed and my list of daily tasks multiplied before my eyes.

To lend some perspective, I started to organise my thoughts and reflections on paper, and soon found that verse provided a structure and rhythm that helped me to understand how I could progress in my own practice and, in my own small way, help families in similar circumstances to my own. The process of writing helped me realise the unique opportunity we have as physicians to gain such an insight into another person's life, and the responsibility of ensuring that we allow patients and their loved ones to tell us a little about the most important things in their lives. Once we understand where a patient has come from, we are in a better position to help them navigate the difficulties they encounter whilst in our care and to ensure that their story can continue in a way that is most congruent with their values. Support from other members of the multidisciplinary team is crucial in providing this holistic care, and the engagement of therapists in rehabilitation, psychologists in support or the chaplain in blessing should not be underestimated.

I wrote this particular poem to give voice to a part of my grandmother's story, highlighting the beautiful things that I remember about her and that I hoped would provide an insight into the person she was and the things that she loved before her illness. To me the process of writing this poem served as a reminder to give all my patients the chance to tell their story, to see past the observation charts and appreciate what truly makes up their lives. Only then can we hope to treat our patients with the compassion and understanding that we hope our own loved ones will experience at the hands of medical professionals; by ensuring that this process starts first with us.

**Evensong**
I remember the ardent goldfinches:
a charm in your camellias,
cheeks sanguine as the Eucharist.
A joyful celestial choir
exuding praise and glory
for long sweet days of Litha.
All Gods and old Gods exalted in unison.

I remember the last slivers of sun
lighting shrinking vestiges
of the rapeseed harvest.
We waded together
along faded paths which led
to the field where horses
rose as monoliths in the fading gloom.

I remember the smell of hot sweet apples;
the spoon crushing vaulted pastry roof,
sinking to the bubbling nave.
Skin still scored and prickling
from circlets of thorns,
the jealous brambles
constraining their inky treasures.
You laughed at their bite and spleen.

I remember the hoary birch branches,
prising the silvery vellum
from the resisting trunk.
Dancing and unravelling,
widdershins around the bole;
sinuous snakes entwining our ankles,
threatening to topple, threatening to fall.

I remember because now you cannot.
A ghost mind astray,
reneging on its past.
Though your smile tells me
that something flickers
far back in your mind:
a fading spark, in the darkness.

## About the author

**Corinne Rowena Dignan** MBChB, is a Foundation Year 2 Doctor who qualified at Keele University Medical School in 2018, and was working at St Cadoc's Hospital, Aneurin Bevan University Health Board, Newport, UK at the time of writing this piece.

